# Case Report: Diabetes mellitus type MODY5 as a feature of 17q12 deletion syndrome with diabetic gastroparesis

**DOI:** 10.3389/fendo.2023.1205431

**Published:** 2023-11-14

**Authors:** Sixu Xin, Xiaomei Zhang

**Affiliations:** Department of Endocrinology, Peking University International Hospital, Beijing, China

**Keywords:** maturity-onset diabetes of the young type 5, MODY 5, chromosome 17q12 deletion syndrome, *HNF1B*, diabetic gastroparesis

## Abstract

**Background:**

Maturity-onset diabetes of the young type 5 (MODY5) is an uncommon, underrecognized condition that can be encountered in several clinical contexts. It is challenging to diagnose because it is considered rare and therefore overlooked in the differential diagnosis. Moreover, no typical clinical features or routine laboratory tests can immediately inform the diagnosis.

**Case presentation:**

We report a 28-year-old man who was once misdiagnosed with type 1 diabetes due to decreased islet function and recurrent diabetic ketosis or ketoacidosis. However, he had intermittent nausea, vomiting, abdominal distension, and abdominal pain 6 months prior. Further examinations revealed agenesis of the dorsal pancreas, complex renal cyst, kidney stone, prostate cyst, hypomagnesaemia, and delayed gastric emptying. Accordingly, whole-exon gene detection was performed, and a heterozygous deletion mutation was identified at [GRCh37 (hg19)] chr17:34842526-36347106 (1.5 Mb, including *HNF1B* gene). The patient was eventually diagnosed with 17q12 deletion syndrome with gastroparesis.

**Conclusion:**

We report a novel case of diabetes mellitus type MODY5 as a feature of 17q12 deletion syndrome caused by a new 17q12 deletion mutation, which will further broaden the genetic mutation spectrum of this condition. With the help of gene detection technology, these findings can assist endocrinologists in making the correct diagnosis of MODY5 or 17q12 deletion syndrome. Additionally, they can formulate an appropriate therapy and conduct genetic screening counseling for their family members to guide and optimize fertility.

## Introduction

Maturity-onset diabetes of the young (MODY) is a monogenic form of diabetes that is inherited in an autosomal dominant manner, and it accounts for approximately 1% –2% of diabetes cases (1). The typical clinical manifestations of MODY are often a family history of three or more generations, disease onset at a young age (before 25 years old), no type 1 diabetes mellitus (T1DM)-related autoantibodies, no need for insulin treatment, and no ketosis tendency. At present, 14 different MODY subtypes that are caused by 14 different pathogenic gene mutations have been identified; MODY5 is due to a mutation in the hepatocyte nuclear factor 1β (*HNF1B*) gene. The incidence of MODY5 is low, accounting for less than 5% of MODY cases (2). The genotype and clinical phenotype of MODY5 are very complex and easily cause misdiagnosis. Almost half of patients diagnosed with MODY5 (*HNF1B* mutation) have a mutation in the form of a whole gene deletion (3). In addition, 17q12 microdeletion syndrome, known as 17q12 deletion syndrome, is a rare chromosomal anomaly caused by the deletion of a small amount of material from a region in the long arm of chromosome 17. It is typified by the deletion of more than 15 genes, including *HNF1B*, resulting in kidney abnormalities, renal cysts, diabetes syndrome [renal cysts and diabetes (RCAD)], and neurodevelopmental or neuropsychiatric disorders (4).

Here, we report a patient who presented with diabetes mellitus (DM)-type MODY5 as a feature of 17q12 deletion syndrome with diabetic gastroparesis (DGP).

## Case presentation

**Figure 1:**
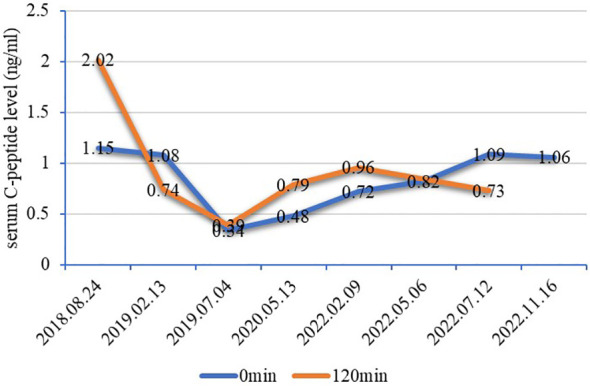
Line chart of serum C-peptide levels. The fasting serum C-peptide level fluctuated between 0.34–1.15 ng/ml (1.1–4.4 ng/ml) and 0.39–2.02 ng/ml for 2 hours after breakfast. There was islet β cell dysfunction but no absolute deficiency of serum C-peptide levels in this patient, which did not conform to the characteristics of T1DM. It is generally believed that C-peptide levels below 200 pmol/L (0.1 ng/ml) after stimulation indicate poor pancreatic function and that C-peptide levels below 600 pmol/L (0.2 ng/ml) after stimulation indicate that the islet function is damaged, which should alert to the possibility of T1DM or monogenic diabetes (5). T1DM, type 1 diabetes mellitus.

**Figure 2:**
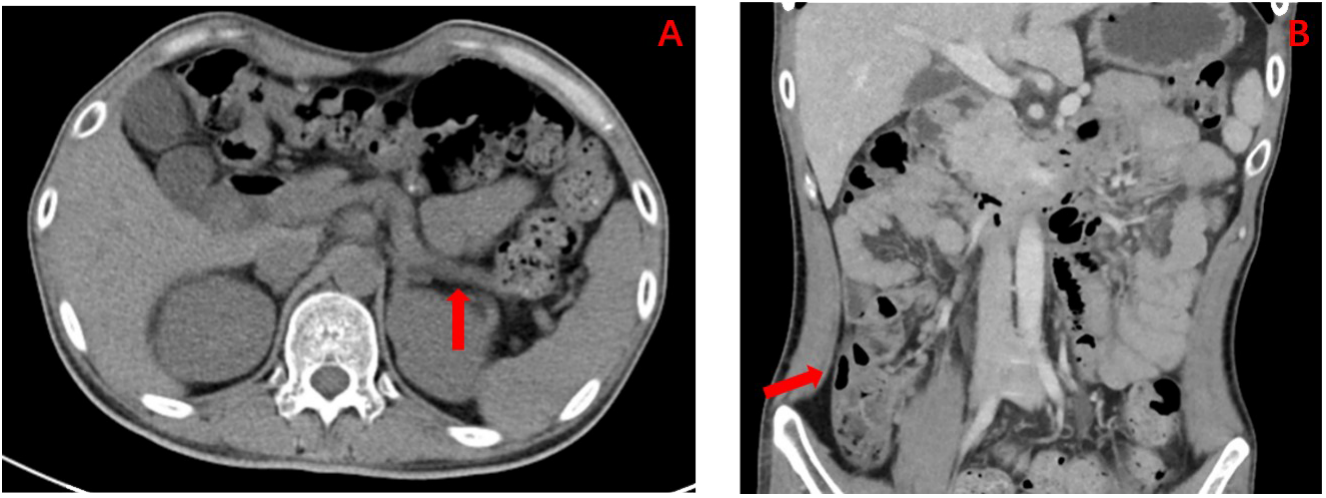
**(A)** Enhanced CT abdomen showing the result of dorsal pancreatic agenesis (as indicated by the red arrows; considering pancreatic developmental variation), an exceedingly rare congenital disease reported in the literature. **(B)** Enhanced CT abdomen showing the result of multiple complex renal cysts (as indicated by the red arrows).

**Figure 3:**
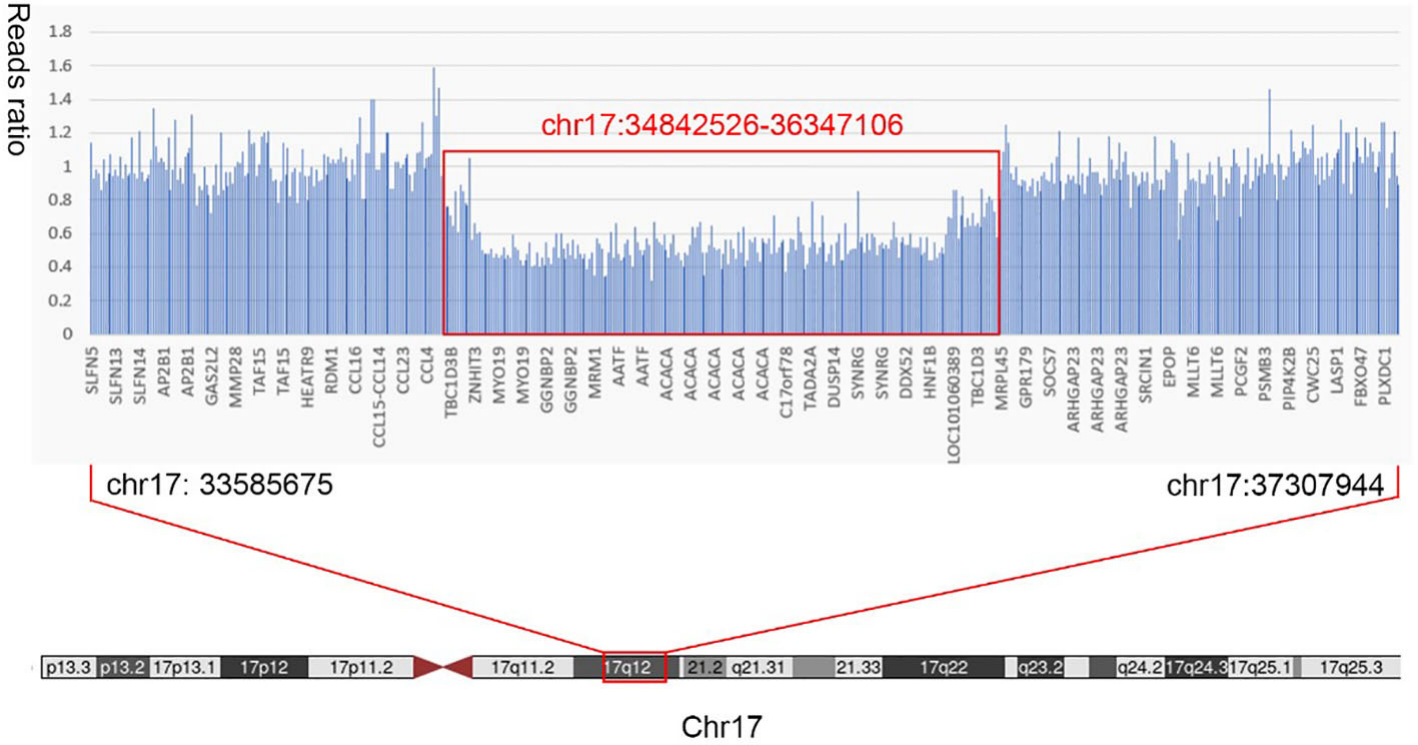
Results of whole-exon gene variation. The patient was suspected to have a heterozygous deletion mutation at chr17:34842526-36347106 (1.5 Mb) that included 24 genes in total: *AATF*, *ACACA*, *C17orf78*, *DDX52*, *DHRS11*, *DUSP14*, *GGNBP2*, *HMGB1P24*, *HNF1B*, *LHX1*, *LHX1-DT*, *MIR2909*, *MIR378J*, *MRM1*, *MYO19*, *PIGW*, *RNA5SP439*, *RNU6-489P*, *SYNRG*, *TADA2A*, *TBC1D3K*, *TBC1D3L*, *YWHAEP7*, and *ZNHIT3*.

The patient, a 28-year-old man, was admitted to the Endocrinology Department of Peking University International Hospital on November 15, 2022, due to “six years of excessive drinking and urination, 6 months of paroxysmal nausea and vomiting”. The patient had symptoms of thirst, polydipsia, and polyuria without inducement 6 years ago and was not diagnosed or treated. The fasting plasma glucose was 8.5 mmol/L (normal reference range is 3.9–6.1 mmol/L) in the posterior examination. Then, he was diagnosed with T1DM after examinations in an external hospital. The patient was given short-acting insulin for three meals and long-acting insulin before bed for anti-hyperglycaemic treatment. He was hospitalized many times for diabetic ketoacidosis (DKA) because of irregular insulin injections. When discharged from the hospital, the patient was given a preprandial subcutaneous injection of insulin lispro and a presleep subcutaneous injection of insulin glargine. The daily insulin consumption was approximately 47–64 units adjusted according to the blood glucose level. Six months prior, he had intermittent nausea and vomiting with no obvious inducement without abdominal pain and diarrhoea. He was diagnosed with diabetic ketosis (DK) by random intravenous plasma glucose with 21.4 mmol/L, arterial blood gas with pH 7.37, and urine ketone body with 3+. The symptoms were alleviated, and ketone bodies were negative after rehydration and insulin supplementation in the emergency department and then our endocrine department. After discharge, he still had intermittent nausea, vomiting, abdominal distension, and abdominal pain, especially after meals. He was given itopride hydrochloride tablets to promote gastrointestinal motility, pancreatin enteric-coated capsules to supplement digestive enzymes, and pinaverium bromide tablets to relieve symptomatic pain. Additionally, psychologists evaluated the patient’s anxiety state and gave duloxetine and oxazepam to relieve anxiety. Unfortunately, the above treatment was not effective. Therefore, the patient himself stopped insulin injections and occasionally measured random peripheral blood glucose > 20 mmol/L. He was then admitted to our department for further diagnosis and treatment. During the course of DM, the patient had no blurred vision, numbness of limbs, cold feeling, acupuncture feeling, sleeve-like feeling, or intermittent claudication. He lost approximately 5 kg of weight in 9 months. For his past history, the patient was diagnosed with a renal cyst, kidney stone, and prostate cyst 3.5 years ago, 8 months ago, and 6 months ago, respectively. He denied a history of pancreatitis or pancreatic surgery. He was born at full term with a birth weight of 2.5 kg without hypoglycaemia. His growth and development are comparable to those of his peers. His mother and other family members did not have a history of DM. Physical examination results were as follows: temperature, 36.2°C; pulse, 72 times/min; respiration, 20 times/min; blood pressure, 140/87 mmHg; height, 175 cm; weight, 50 kg; BMI, 16.33 kg/m^2^; waistline, 65 cm; hip, 77 cm; and waist-to-hip ratio, 0.84. He did not have a Cushing appearance. He had clear breath sounds in both lungs, no obvious dry and wet rales, regular heart rhythm, no murmur, and additional heart sounds in the auscultation area of each valve. He had boat-shaped abdomen, soft whole abdomen, mild tenderness in the upper abdomen, no rebound pain and muscle tension, normal bowel sounds, no oedema in both lower limbs, normal pulsation of bilateral dorsalis pedis arteries, normal sense of pain, temperature, and vibration, and a negative 10 g elastic wire test on both sides. Laboratory examination revealed that the venous fasting plasma glucose was 26.3 mmol/L, urine glucose 3+, and urine ketone body +. The arterial blood gas analysis was as follows: pH 7.46 (7.35–7.45), PaO_2_ 81 mmHg (80–100 mmHg), PaCO_2_ 48 mmHg (35–45 mmHg), HCO3 ^−^ 35.0 mmol/L (22–27 mmol/L), BE-b 10.5 mmol/L (−3.0 to 3.0 mmol/L), venous serum potassium ion 3.5 mmol/L (3.5–5.5 mmol/L), sodium ion 134 mmol/L (137–147 mmol/L), chloride ion 93 mmol/L (99–110 mmol/L), glycosylated haemoglobin 11.2% (4.0%–6.0%), and fasting serum C-peptide level fluctuated between 0.34–1.15 ng/ml (1.1–4.4 ng/ml) and 0.39–2.02 ng/ml 2 hours after breakfast (**Figure 1**). The patient was negative for glutamic acid decarboxylase antibody (GADA), islet cell antibody (ICA), and insulin autoantibody (IAA). The serum magnesium level fluctuated between 0.39 and 0.71 mmol/L (0.75–1.02 mmol/L). His liver function, glomerular filtration rate, serum lipids, uric acid, calcium, phosphorus, parathyroid hormone, and thyroid function were all within the normal range. The complications of diabetes were examined. No diabetic retinopathy was found in fundus photography, but cataracts were found in the right eye. The successive urinary microalbumin/creatinine ratio (UACR) was measured three times, and the values were 22.32 mg/g, 16.7 mg/g, and 15.85 mg/g (0–30 mg/g). Colour Doppler ultrasound of the carotid artery and lower limb artery showed no atherosclerotic plaque formation. Measurements of pulse wave velocity (PWV), ankle–brachial index (ABI), and quantitative sensory disturbance were normal. Digestive system diseases were evaluated. The enhanced CT scan of the abdomen showed agenesis of the dorsal pancreas (ADP) (considering pancreatic developmental variation), complex cysts of both kidneys, and small stones in the right kidney (**Figure 2**). An abdominal dynamic contrast-enhanced magnetic resonance scan did not show pancreatic duct dilation. Gastric emptying imaging showed that the gastric half-emptying time of semisolid food was approximately 80.53 min (37.25 ± 15.7 min). The tumor markers of the digestive tract, serum amylase, and lipase were normal. Gastroscopy showed chronic non-atrophic gastritis with bile reflux and a positive urease *Helicobacter pylori* (HP) test. Enteroscopy showed that the intestinal preparation was poor and that the mucosa below the sigmoid colon was normal. No abnormality was found in the upright abdominal plain film radiography and the ultrasound of the superior and inferior mesenteric arteries. The characteristics of this case are summarized as follows: 1) young onset of illness with a primary diagnosis of T1DM and therapy with long-term insulin replacement; 2) no family history of DM; 3) islet β cell dysfunction, but no absolute deficiency was observed; 4) negative for diabetes-related antibodies; and 5) abnormal development of multiple organs. Therefore, the diagnosis of T1DM was challenged. Specific types of DM could not be excluded. After informed consent of the patient was obtained, a peripheral blood sample was taken to Beijing Hope Group Biotechnology Co., Ltd., for examination. First, DNA interruption was performed, and a library was prepared. Then, DNA sample capture and PCR amplification were performed. Finally, the captured DNA samples were submitted for high-throughput sequencing. After the sequencing data were evaluated by Illumina Sequence Control Software (SCS) and qualified, data reading and bioinformatics analysis were performed. The results showed that the patient was suspected to have a heterozygous deletion mutation at [GRCh37 (hg19)] chr17:34842526-36347106 (1.5 Mb) that included 24 genes in total: *AATF*, *ACACA*, *C17orf78*, *DDX52*, *DHRS11*, *DUSP14*, *GGNBP2*, *HMGB1P24*, *HNF1B*, *LHX1*, *LHX1-DT*, *MIR2909*, *MIR378J*, *MRM1*, *MYO19*, *PIGW*, *RNA5SP439*, *RNU6-489P*, *SYNRG*, *TADA2A*, *TBC1D3K*, *TBC1D3L*, *YWHAEP7*, and *ZNHIT3* (**Figure 3**). This region contained the complete 17q12 recurrent region (including *HNF1B* gene), which is not enough to cause a disease with the confirmed single dose. According to the results of gene detection, the diagnosis was revised to 17q12 deletion syndrome. Considering the findings of poor long-term blood glucose control, significantly delayed gastric emptying time, and the digestive tract history, the patient was considered to have DGP. The treatment plan was as follows: 1) diet guidance of low-fat diabetes soft food or semiliquid food and multiple meals with small amounts for each, 2) insulin subcutaneous injection for glucose-lowering treatment, 3) mosapride citrate tablets for promoting total gastrointestinal motility, and 4) pancreatin enteric capsules for supplementing pancreatin. Treatment results and follow-up indicated that the patient’s blood glucose fluctuation was reduced, with his fasting blood glucose fluctuating between 4 and 8 mmol/L and his 2-hour postprandial glucose fluctuating between 6 and 11 mmol/L. No symptomatic hypoglycaemia events occurred. His abdominal symptoms were significantly alleviated. Afterward, he did not seek medical attention due to unbearable abdominal pain (**Table 1**).

**Table 1:** The timeline with relevant data from the episode of care.

Date		Symptoms	Examinations	Diagnosis	Treatments	Effects
2016 onset		thirst, polydipsia, polyuria	FPG: 8.5mmol/L, other results unavailable	T1DM	subcutaneous injection of short-acting insulin for three meals and long-acting insulin before bed (47-64 units per day)	repeated emergency or hospitalization treatment due to DK/DKA
2022.05.06		intermittent nausea, vomiting, abdominal distension and pain, diarrhea	FPG: 21.4mmol/L, HbA1c: 8.5%, fasting C-peptide: 0.82ng/ml, GADA: negative, ICA: negative, IAA: negative, urine glucose: 4+, urine ketone: 3+, arterial blood pH: 7.37, serum magnesium: 0.46mmol/L	DK	intravenous fluid infusion and insulin supplementation, itopride hydrochloride tablets, pancreatin enteric-coated capsules, pinaverium bromide tablets, duloxetine and oxazepam	peripheral blood sugar <10 mmol/L, urine glucose: 3+, urine ketone: +, no significant improvement in digestive system symptoms
2022.05.16- 2022.08.22	2022.05.16	unresolved digestive symptoms including intermittent nausea, vomiting, abdominal distension and pain, diarrhea, aggravated after meals	gastroscopy: chronic non atrophic gastritis with bile reflux and a positive urease HP test	chronic non atrophic gastritis, Helicobacter pylori infection	Omeprazole Enteric-coated Capsules, Minocycline Hydrochloride Tablets, Colloidal Bismuth Pectin Capsules, Mosapride Citrate TabletsTrimebutine, Maleate Tablets, Berberine Hydrochloride Tablets, Montmorillonite Powder, Bacillus Licheniformis Capsule, Live Combined Bifidobacterium, Lactobacillus And Enterococcus Capsules, Pinaverium Bromide Tablets, Oryz-Aspergillus Enzyme and Pancreatin Tablet	poor response to treatment
	2022.07.09		enhanced CT scan of the abdomen: agenesis of the dorsal pancreas, complex cysts of both kidneys and small stones in the right kidney	ADP, complex renal cysts in both kidneys, right kidney stone		
	2022.07.22		ultrasound of the superior and inferior mesenteric arteries	Normal		
	2022.08.08		enteroscopy and upright abdominal plain film radiography	Normal		
	2022.08.10		MRCP	No dilation of pancreatic duct		
	2022.08.21		blood testing: liver function, CEA, CA19-9, CA72-4, AFP, amylase, lipase	Normal		
	2022.08.22		gastric emptying imaging: positive ^a^	Gastroparesis	diet guidance of low-fat diabetes soft food or semiliquid food and multiple meals with small amounts for each	improved digestive system symptoms
2022.08.31			whole exon gene detection (plasma) ^b^	17q12 Deletion Syndrome	insulin subcutaneous injection	FPG: 4-8 mmol/L, 2h-PPG: 6-11 mmol/L

FPG, fasting plasma glucose; T1DM, type 1 diabetes mellitus; DK, diabetic ketosis; DKA, diabetic ketoacidosis; GADA, glutamic acid decarboxylase antibody; IAA, insulin autoantibody; ICA, islet cell antibody; HbA1c, glycosylated haemoglobin; CT, computed tomography; ADP, genesis of the dorsal pancreas; MRCP, magnetic resonance cholangiopancreatography; CEA, carcinoembryonic antigen; CA19-9, cancer antigen 19-9; CA72-4, cancer antigen 72-4; AFP, alpha-fetoprotein; 2h-PPG, 2-hour postprandial glucose.

a Gastric emptying imaging showed that the gastric half-emptying time of semisolid food was approximately 80.53 min (37.25 ± 15.7 min).

b The results of plasma whole-exon gene detection showed that the patient was suspected to have a heterozygous deletion mutation at [GRCh37 (hg19)] chr17:34842526-36347106 (1.5 Mb). This region contained the complete 17q12 recurrent region (including HNF1B gene).

## Discussion

Here, we report a novel case of diabetes mellitus type MODY5 as a feature of 17q12 deletion syndrome caused by a new 17q12 deletion mutation (including *HNF1B* gene) that has not been reported yet worldwide. 17q12 microdeletion syndrome, also known as 17q12 deletion syndrome, is a rare chromosomal anomaly caused by the deletion of a small amount of material from a region in the long arm of chromosome 17; this syndrome has an estimated prevalence of 1.6 per 100,000 citizens, with high penetrance and variable expressivity (6). Because the exogenic region on 17q12 is encompassed by segmental duplications, all patients with this syndrome exhibit the same unique genetic sequence of 1.4 Mb, including *HNF1B*, *ACACA*, and *LHX1* genes (7). It is typified by the deletion of more than 15 genes, including *HNF1B*, and results in kidney abnormalities, RCAD, and neurodevelopmental or neuropsychiatric disorders (4). This patient presented with kidney abnormalities and RCAD.

As described for intragenic *HNF1B* mutations, DM represents a frequent feature of 17q12 deletion syndrome and affects 63% of these patients (8). Although the association of DM and renal dysfunction in patients with *HNF1B* mutations is commonly known as RCAD, the frequent overlap of *HNF1B* mutations with 17q12 deletion syndrome is often underrecognized. Laffargue et al. (3) reported an association between *HNF1B* deletion and 17q12 deletion syndrome in virtually all cases. ADP is a very rare pancreatic developmental anomaly. During embryonic development, the dorsal pancreatic bud develops into the dorsal pancreas, which forms a small part of the pancreatic head, body, and tail. Less than 100 cases of ADP have been reported worldwide (9). There are two subtypes of ADP, complete and partial, in which the body or tail of the pancreas is underdeveloped or underdeveloped. In this case, the tail of the pancreas was missing, indicating partial dorsal pancreatic hypoplasia. The latest research has found that homeotic genes *GATA6* and *GATA4*, as well as human *HNF1B* gene, are involved in the regulation of pancreatic development. Human *HNF1B* gene belongs to the homeobox-containing family of transcription factors and is located on chromosome 17. It has regulatory effects on the morphological development of the pancreas and the differentiation of pancreatic endocrine cells (10–12). Because most islet cells are located in the tail of the pancreas, ADP usually causes insufficient islet cells, leading to the occurrence and development of diabetes. It also participates in the development of many important organs, including the kidney, liver, and reproductive system, and plays a central role in maintaining the normal function of organs (13). This gene is also the pathogenic gene of MODY5. The first *HNF1B* mutation with manifestation of MODY5 was described in 1997 (14). Since then, a great variety of clinical phenotypes have been described. The characteristic clinical manifestations of MODY5 include early-onset diabetes, kidney disease, pancreatic atrophy, pancreatic exocrine dysfunction, liver dysfunction, hypomagnesaemia, and urogenital abnormalities. Our patient presented with typical polycystic kidney manifestations, ADP, kidney stones, and prostate cysts, which were consistent with previous reports (15, 16). Although studies have shown that patients with mutations might have worse kidney damage than those with gene deletions (15), kidney damage was not present in this patient. These characteristics may exist without a family history because *de novo* mutations reportedly occur relatively frequently, as they are seen in 50%–60% of patients with *HNF1B* gene abnormalities (17, 18). This is believed to be the case. Currently, more than 230 *HNF1B* gene abnormalities have been identified, of which the incidence of gene deletions is higher than that of point mutations (19). However, most of them are heterozygous deletions. Large segment deletions or duplications of exons are extremely rare (15). Because almost half of patients diagnosed with MODY5 (*HNF1B* mutation) have a mutation in the form of a whole gene deletion, which is the most common cause of MODY5 (3), all patients with suspected MODY5 should be examined for common clinical features of 17q12 deletion syndrome to perform specific microarray testing of the deletion on chromosome 17q12. However, it should be noted that *de novo* mutations are responsible for 70% of 17q12 deletions (8). Thus, the absence of DM and other typical clinical features in their parents and other relatives could not exclude 17q12 deletion syndrome as a possible diagnosis. The risk for inheritance of this deletion in the offspring of affected patients is 50% (8). Consequently, all patients suspected or diagnosed with 17q12 deletion syndrome should be offered genetic counseling.

As mentioned earlier, ketoacidosis is rare at the time of diagnosis in patients with MODY5 (15). Studies have shown that there are differences in the function of islet cells in patients with MODY5 of different ethnic groups. Japanese individuals usually exhibit β cell dysfunction, while Caucasian individuals exhibit hyperinsulinaemia and insulin resistance (18). It is worth considering that the daily insulin dosage of this patient was 47–64 U, and the serum C-peptide level did not indicate an absolute lack of pancreatic islet function. However, he had recurring DK or DKA. Shenghui Ge et al. (20) reported possible reasons for this. First, there is obvious heterogeneity in MODY5 patients, and the clinical manifestations are different among different patients. Second, DK is a rare manifestation of MODY5 that might not have received much attention in previous studies (21). Finally, there might be a cumulative effect in the development of diabetes and DK, which occur when the disturbance of glucose metabolism caused by the mutation reaches a certain level. It has been reported that *HNF1B* mutations can also affect Na-Cl cotransporter function in the distal convoluted tube, leading to hypokalaemia, hypomagnesaemia, and metabolic alkalosis (22, 23). In the present case, hypomagnesaemia caused by Na-Cl cotransporter dysfunction seems to predominate over mild DKA caused by insulin deficiency in MODY5. In addition, DK typically manifests with acidaemia due to an accumulation of acidic ketone bodies. However, it can present as alkalaemia under certain but limited conditions, including vomiting and the use of diuretics. In this case, before the onset of nausea, vomiting, and abdominal pain, he had recurring DK and DKA. Thus, the above aetiological mechanisms are not suitable for this case. In addition, DKA resolved gradually after insulin therapy, but the abdominal pain, nausea, and vomiting continued. Although endocrine dysfunction constitutes one of the defining clinical manifestations of 17q12 deletion syndrome, impairment of exocrine pancreatic function is highly variable. Dubois-Laforgue et al. (15) reported exocrine pancreas dysfunction in 29 of 38 investigated patients (76%). Natascha Roehlen et al. (7) also revealed a frequency of 69% in all reported cases of *HNF1B* deletion. Tian Yang et al. (24) reviewed 22 cases of ADP with dorsal pancreatic hypoplasia. Although the majority of ADP patients were asymptomatic, abdominal pain was the most commonly reported symptom. Regrettably, the faecal elastase level could not be measured for our patient. After the experimental administration of pancreatin supplement, the patient’s abdominal pain did not improve significantly. He also had digestive tract symptoms such as nausea, vomiting, and abdominal distension, which were significantly worse after eating meals. Considering the long-term poor control of blood glucose, the gastric emptying time was significantly delayed. There was reason to believe that DGP was also involved in the occurrence of abdominal pain. Therefore, a low-fat diet was recommended, and pancreatic enzyme supplements and mosapride citrate were given with meals to facilitate the digestive process. Abdominal pain and other gastrointestinal symptoms improved gradually.

## Conclusion

In summary, we first reported a novel case of diabetes mellitus type MODY5 as a feature of 17q12 deletion syndrome with DGP. The deletion mutation at chr17: 34842526-36347106 has not yet been reported, which will further broaden the gene mutation spectrum of 17q12 deletion syndrome. It is worth noting that, as a manifestation of 17q12 deletion syndrome, MODY5 patients may have recurrent DK or DKA or no family history of DM. For patients with early-onset diabetes who are negative for insulin-related antibodies and have hypomagnesaemia and abnormalities of the kidney and pancreas, we should be alert to MODY5. Gene detection technology can assist endocrinologists in correctly diagnosing MODY5 or 17q12 deletion syndrome. However, it can also formulate appropriate therapy and conduct genetic screening counseling for their family members to guide and optimize fertility. However, this case report still has certain limitations. 1) Unfortunately, we were unable to obtain the patient’s genetic pedigree. 2) As this is a case of 17q12 syndrome, it is regrettable that the patient’s reproductive tract structure and function were not tested to comprehensively track the characteristics of their case. 3) The number of cases should be increased, and their clinical commonalities should be summarized to better understand the disease.

## Data availability statement

The original contributions presented in the study are included in the article/supplementary material. Further inquiries can be directed to the corresponding author.

## Ethics statement

The studies involving humans were approved by Biomedical Ethics Committee of Peking University International Hospital. The studies were conducted in accordance with the local legislation and institutional requirements. The participants provided their written informed consent to participate in this study. Written informed consent was obtained from the individual(s) for the publication of any potentially identifiable images or data included in this article.

## Author contributions

SX: Data extraction, data analysis, essay writing, and paper submission. XZ: Critical revision and paper submission. All authors contributed to the article and approved the submitted version.
